# Height and body mass index trajectories from 1975 to 2015 and prevalence of stunting, underweight and obesity in 2016 among children in Chinese cities: findings from five rounds of a national survey

**DOI:** 10.1007/s12519-023-00747-1

**Published:** 2023-08-17

**Authors:** Xin-Nan Zong, Hui Li, Ya-Qin Zhang

**Affiliations:** Department of Growth and Development, Capital Institute of Pediatrics, Beijing 100020, China

**Keywords:** Body mass index, Children, Height, Obesity, Stunting

## Abstract

**Background:**

A more comprehensive assessment of growth and nutrition in children is required in China due to rapid socioeconomic processes. We aimed to investigate height and body mass index (BMI) trajectories from 1975 to 2015 and the prevalence of stunting and obesity in 2016 among children in Chinese cities.

**Methods:**

A total of 904,263 children from birth to 6.9 years were collected from a series of nationally representative surveys in China. Height and BMI trajectories and prevalence of stunting, underweight, overweight and obesity were assessed.

**Results:**

The average height level of Chinese urban children under 7 years presented a positive secular trend from 1975 to 2015; however, a slowing tendency occurred in 2005‒2015. An apparent increase was observed at the 5th, 50th and 95th percentiles of BMI in urban children aged 3 years and older, with a more prominent increase at the 95th percentile. The total prevalence of stunting and underweight under 7 years was 1.4% and 2.0%, respectively. The total prevalence of overweight and obesity under 7 years was 12.6% and 4.3%, respectively, with 12.7% and 4.9% for boys, 12.6% and 3.6% for girls, 12.1% and 4.0% in urban areas and 13.1% and 4.5% in suburban rural areas.

**Conclusions:**

The average height level of Chinese urban children has reached World Health Organization child growth standards since 2005 and presented a slowing tendency in secular trend in 2005‒2015. More attention and efforts and public health interventions should be urgently made to combat overweight and obesity among preschool children.

**Video Abstract**

**Supplementary Information:**

The online version contains supplementary material available at 10.1007/s12519-023-00747-1.

## Introduction

The growth and nutrition of children is one of the main markers of socioeconomic development and civilizational progress in a population. Regular monitoring and assessment of children’s growth and nutrition is essential and conducive to the formulation and revision of children’s health and nutrition strategies and rational investment and allocation of health resources. The Chinese government attaches great importance to the growth, development and nutrition of children. It launched a national special survey entitled “the National Survey on Physical Growth and Development of Children (NSPGDC)” in nine cities in China in 1975, and this national survey has continued to the present day [1, 2]. The NSPGDC is the earliest, large-scale, population-based nationally representative special survey on the growth and development of children under 7 years old, and the NSPGDC and its subprojects are primary data sources for developing national health strategies and national growth standards for Chinese children [3–5].

China transitioned from a low-income to an upper middle-income country along with the sustained and rapid development of the social economy in the past few decades. An overall trend, such as decreasing under-nutrition and increasing over-nutrition was reported among children and adolescents aged 7–18 years in China between 1985 and 2014 [6, 7]. However, a more comprehensive analysis and assessment report in young children is still inadequate or even lacking in China with rapid socioeconomic processes, which is not conducive to evidence-based revision and improvement of children’s health and nutrition strategies.

Using five rounds of the NSPGDC [1, 2, 8–10] and one of its subprojects [11], we aimed to investigate 40-year trends in height and body mass index (BMI) trajectories from 1975 to 2015 and the prevalence of stunting, underweight, overweight and obesity in 2016 among children under 7 years old in Chinese cities. In addition, we also examined the effect of some economic or sociodemographic variables on children’s growth and nutrition. Our spatiotemporal analysis may be useful for the revision and refinement of children’s health and nutrition strategies for China as well as other rapidly developing countries or regions.

## Methods

### Data source

Data were derived from the NSPGDC in nine cities of China [1, 2, 8–10], which was a repeated cross-sectional national special survey undertaken since 1975, once every 10 years, and a subproject of the NSPGDC, namely, the National Epidemiological Survey on Simple Obesity in Childhood (NESSOC) in the same nine cities [11]. To exhibit a more complete picture of the growth and nutrition of Chinese children, we used all data of the series of the NSPGDC from 1975 to 2015 to exhibit secular trends in height and BMI trajectories and the latest data of the NESSOC in 2016 to examine the updated prevalence of stunting, underweight, overweight and obesity among children under 7 years old in Chinese cities.

The NSPGDC was carried out in five rounds in nine cities of China in 1975, 1985, 1995, 2005 and 2015. Of these nine cities, Beijing and Shanghai are province-level municipalities that have almost the highest level of socioeconomic development in China, and the other seven cities are provincial capital cities that have almost the highest level of socioeconomic development in their respective provinces, including Harbin (Heilongjiang’s provincial capital), Xi’an (Shaanxi), Nanjing (Jiangsu), Wuhan (Hubei), Guangzhou (Guangdong), Fuzhou (Fujian) and Kunming (Yunnan). On the other hand, these nine cities covered all seven geographical regions of China, including East China (Shanghai, Nanjing, Fuzhou), South China (Guangzhou), North China (Beijing), Central China (Wuhan), Southwest China (Kunming), Northwest China (Xi’an), and Northeast China (Harbin). The NSPGDC used stratified cluster sampling according to urban/suburban rural areas and administrative districts in each survey city. Children under 3 years old were from the street community, and children aged 3 to 6.9 years were from kindergarten. Urban children are determined as permanently living in urban areas in the surveyed city, or these children moved into the surveyed city from other large cities and lived for more than two-thirds of the children’s age; and suburban rural children, as either one or both parents are farmers and children are brought up in the suburban rural area (surrounding the surveyed city). Children under 7 years old are classified into 22 age groups: newborn to 3 days, monthly for 1 to 5 months, bimonthly for 6 to 11 months, tri-monthly for 12 to 23 months, half-yearly for 2 to 5.9 years, and yearly for 6 to 6.9 years. The classification of age groups follows the criteria of ‘‘low limit’’; for example, 3 months represents children aged 3.0–3.9 months. Each sex-age subgroup consists of about 200 children in urban/rural areas of each survey city. About 1800 children were included in each age subgroup for urban boys, urban girls, rural boys and rural girls. Exclusion criteria were temporary residents, premature birth, birth weight below 2.5 kg, multiple births, acute illness within a month, chronic illness, and obvious malnourishment and physical handicap.

The NESSOC was one of the subprojects of the NSPGDC and the most representative epidemiological special survey on childhood obesity in China [11, 12], and the surveyed cities and sampling method were the same as the NSPGDC. One or more districts were selected as the study units in each survey city, and the estimated numbers of children under 7 years old were more than 10 thousand, with about half in urban areas and half in rural areas. Children below 3 years were selected from the street community, and children aged 3 to 6.9 years were selected from kindergarten. The survey number of communities and kindergartens was estimated based on the total number of children under 7 years old and the age distribution of children in the selected districts of each survey city. The participation proportion of the study subjects was not less than 95% in the selected communities and kindergartens, and the total number of participants was not less than 10 thousand in each survey city.

### Sample size

A total of 904,263 children under 7 years were included in this present study, with 793,772 children from five rounds of the NSPGDC between 1975 and 2015 and 110,491 children from the NESSOC in 2016. Table 1 shows the detailed sample sizes by gender, urban/rural area, and survey year.

**Table 1:** Sample sizes by gender, urban/suburban rural area, and survey year

Survey year	Boys		Girls		Total
	Urban	Rural	Urban	Rural	
NSPGDC					
1975	47,844	45,333	46,652	43,158	182,987
1985	39,600	36,840	39,594	36,840	152,874
1995	39,590	39,301	39,564	38,907	157,362
2005	34,901	34,650	34,859	34,365	138,775
2015	41,990	39,161	41,638	38,985	161,774
NESSOC					
2016	29,055	28,863	26,465	26,108	110,491

*NSPGDC* National Survey on Physical Growth and Development of Children, *NESSOC* National Epidemiological Survey on Simple Obesity in Childhood

### Anthropometric measurements

Using unified measuring tools/instruments in a standardized way by specially trained technicians or nurses [2, 13], weight was measured with children wearing the lightest vest, shorts or underwear by newborn scales (accurate to 10 g) for newborns, and lever scales (accurate to 50 g, in 1975–2005) or electronic scales (T-scale M303, accurate to 50 g, in 2015) for children aged 1 month to 6.9 years, and height (not in shoes) was measured as recumbent length with a horizontal infantometer (accurate to 0.1 cm) for children under 3 years old and as standing height with a metal column height measuring device (accurate to 0.1 cm) for children aged 3 to 6.9 years in 1975‒2015. All height measurements were recorded to the nearest 0.1 cm. Measurement errors of weight and height were not more than 0.05 kg or 0.5 cm between two repeated measurements.

### Quality control

The NSPGDC and its subproject (NESSOC) were undertaken in nine cities of China. The steering committee was located in Beijing (capital of China), and the nine coordinating subgroups in each survey city worked simultaneously following a uniform program, organizing and training local survey teams, carrying out the measurements and checking the questionnaires under the direction and supervision of the Beijing steering committee. All the field investigators participated in rigorous training and passed professional examinations before starting the field investigation. Unified measuring tools/instruments were equipped for each field site of each survey city. The field investigation started in May and finished in October of the same year. All physical measurements were carried out at least 1 hour after a meal between approximately 8 a.m. and 4 p.m. The questionnaire design, questionnaire entry and data verification were completed by the Beijing steering committee.

### Economic or sociodemographic data

Data on gross domestic product (GDP) per capita, Engel’s coefficient, health professionals including doctors and nurses, and under-5 and infant mortality rates between 1978 and 2020 were obtained from the China Statistical Yearbook 2022 [14].

### Statistical analysis

BMI was calculated as weight (kg) divided by the square of length/height (m). A 40-year change in height from 1975 to 2015 was examined, but only a 30-year change in BMI from 1985 to 2015 was analyzed due to the scarcity of the raw dataset in 1975, and BMI was not calculated and documented in 1975, as BMI was not widely used. We defined prevalence of stunting as < − 2 standard deviations (SD) from median for length/height-for-age, underweight as < − 2 SD, overweight as ≥ + 1 SD but < + 2 SD, and obesity as ≥ + 2 SD from median for BMI-for-age based on the China growth standards for 0–18 years [4] or the World Health Organization (WHO) growth standards for 0–5 years and the WHO growth references for 5–19 years [15, 16]. A comparable definition of the prevalence according to the same cutoffs when using the China and WHO criteria may be more feasible and reasonable for the under-7-year data in this present study. Differences in the prevalence based on the China and WHO criteria were tested by McNemar’s test. A two-sided *P* value of less than 0.05 was considered statistically significant. Data analysis was performed in SAS v9.4 (SAS Institute, Cary, North Carolina).

## Results

### Height trajectory change from 1975 to 2015

**Fig. 1:**
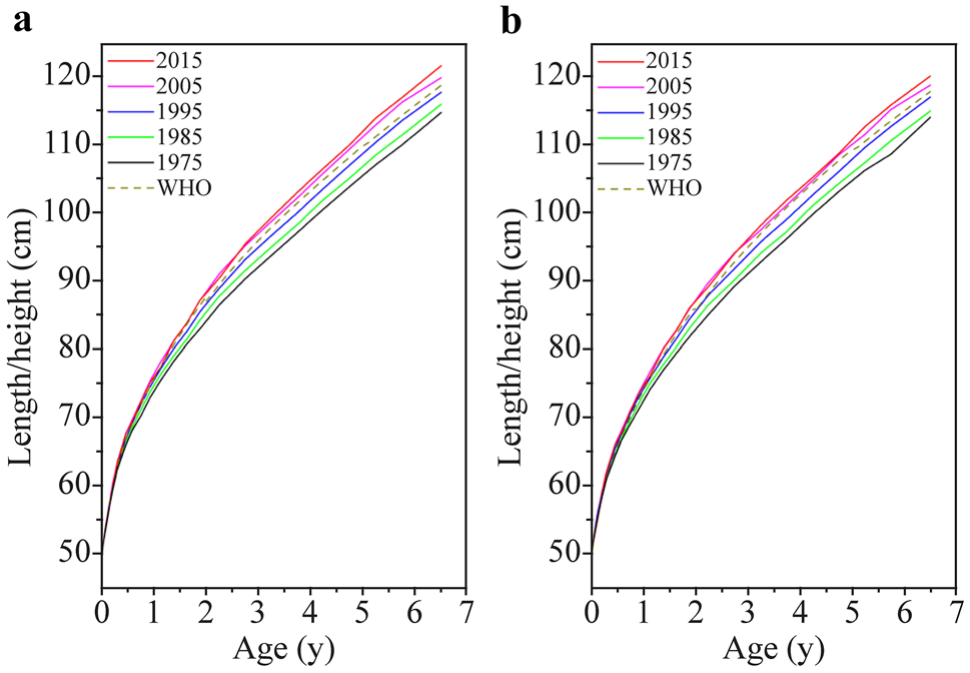
Secular trends in median height in urban children from 1975 to 2015 and comparisons with the World Health Organization standards/references. **a** Boys; **b** girls

The average height level of Chinese urban children under 7 years presented a positive secular trend between 1975 and 2015; however, a slowing tendency occurred in 2005‒2015 (Fig. 1), with a total of 5.8 cm increments in 1975‒2015 (1.3 cm in 1975‒1985, 1.7 cm in 1985‒1995, 2.2 cm in 1995‒2005 and 0.7 cm in 2005‒2015) for boys aged 3 years and a total of 5.6 cm increments (1.2 cm, 1.8 cm, 1.9 cm and 0.7 cm, respectively) for girls. Compared with WHO standards/references, the height level of Chinese urban children under 7 years has undergone a changing process from falling behind in 1995 and before to reaching in 2005 and to outstripping in 2015.

**Fig. 2:**
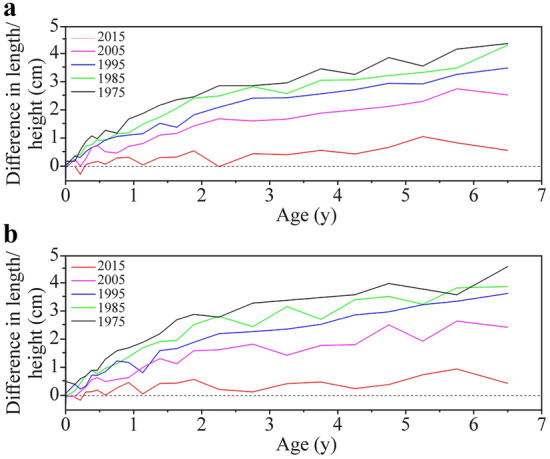
Secular trends in urban‒rural differences in median height in children, 1975–2015. **a** Boys; **b** girls

The urban‒rural differences in average height showed a narrowing tendency between 1975 and 2015, with a sharp narrowing gap in 2005‒2015, and the differences in 2015 were less than 0.5 cm in most of the age groups (Fig. 2). For example, the urban‒rural differences were 3.3 cm in 1975, 2.9 cm in 1985, 2.5 cm in 1995, 1.8 cm in 2005 and 0.5 cm in 2015 for boys aged 3 years and 3.5 cm, 3.0 cm, 2.5 cm, 1.6 cm and 0.4 cm for girls, respectively.

### Body mass index trajectory change from 1985 to 2015

**Fig. 3:**
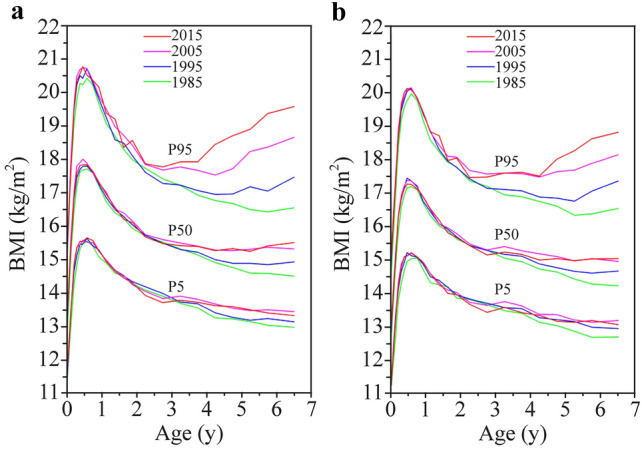
Secular trends in body mass index (BMI) by the 5th, 50th and 95th percentiles in urban children, 1985–2015. **a** Boys; **b** girls

**Fig. 4:**
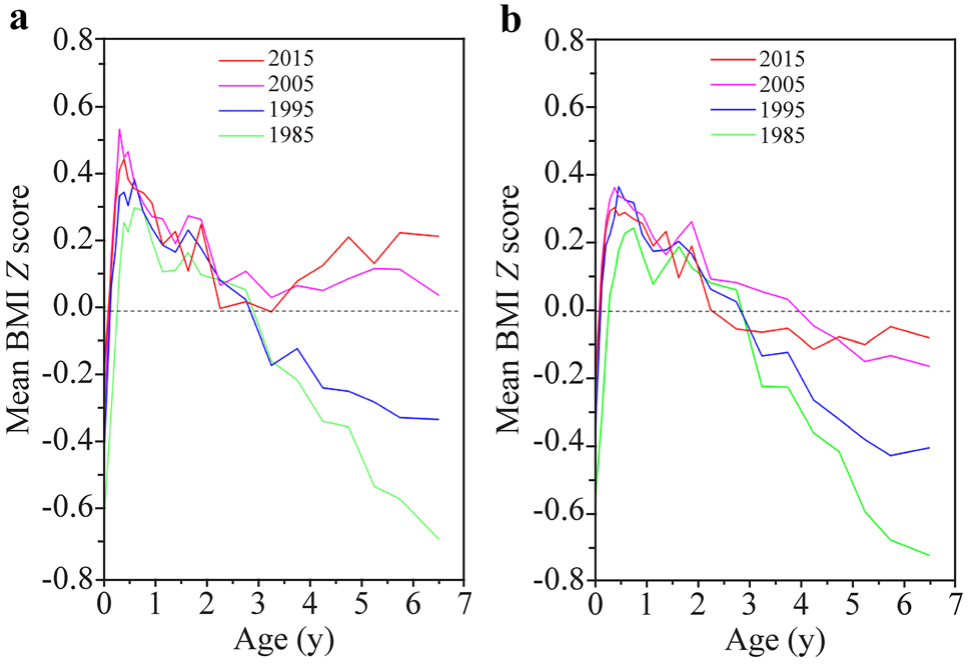
Secular trends in mean body mass index (BMI) *Z* score (calculated as World Health Organization criteria) in urban children, 1985–2015. **a** Boys; **b** girls

An apparent increase was observed at the 5th, 50th and 95th percentiles of BMI in urban children aged 3 years and older, with a more prominent increase at the 95th percentile (Fig. 3), for example, a total of 2.7 kg/m^2^ increments at the 95th percentile during 1985–2015 (0.7 kg/m^2^ in 1985–1995, 1.2 kg/m^2^ in 1995–2005 and 0.8 kg/m^2^ in 2005–2015) for boys aged 5 years and a total of 2.2 kg/m^2^ increments (0.6 kg/m^2^, 0.9 kg/m^2^ and 0.7 kg/m^2^, respectively) for girls. Furthermore, the nutritional level of urban children aged 3–6.9 years was higher for boys but lower for girls than WHO standards/references in 2015 (Fig. 4).

### Stunting prevalence in 2016

The total prevalence of stunting based on the Chinese criteria was 1.4% (Table 2). The stunting prevalence was 1.4% for boys and 1.4% for girls, ranging from 0.7% to 1.9% among different age groups, was 1.2% in urban areas and 1.6% in rural areas, and varied from the lowest 0.3% in Nanjing to the highest 4.6% in Guangzhou. The stunting prevalence was lower based on the WHO criteria than based on the Chinese criteria in all the corresponding examined groups (Table 2).

**Table 2:** Prevalence of stunting in children under 7 years in nine cities of China based on the China and WHO criteria

Variables	Sample size	China criteria	WHO criteria	Chi-square	*P*
Total	110,499	1545 (1.4)	743 (0.7)	756.60	< 0.001
Gender					
Boys	57,921	827 (1.4)	458 (0.8)	326.32	< 0.001
Girls	52,578	718 (1.4)	285 (0.5)	431.00	< 0.001
Age (y)					
1–11 mon	17,328	226 (1.3)	167 (1.0)	48.75	< 0.001
1–1.9	16,330	164 (1.0)	137 (0.8)	10.73	0.001
2–2.9	13,669	102 (0.7)	88 (0.6)	12.07	0.001
3–3.9	15,454	187 (1.2)	91 (0.6)	94.01	< 0.001
4–4.9	19,932	380 (1.9)	142 (0.7)	236.00	< 0.001
5–5.9	17,009	311 (1.8)	75 (0.4)	234.00	< 0.001
6–6.9	10,777	175 (1.6)	43 (0.4)	130.00	< 0.001
Area					
Urban	55,524	649 (1.2)	301 (0.5)	325.43	< 0.001
Rural	54,975	896 (1.6)	442 (0.8)	429.30	< 0.001
Region/cities					
Beijing	15,649	101 (0.6)	42 (0.3)	53.39	< 0.001
Harbin	15,270	119 (0.8)	68 (0.4)	47.17	< 0.001
Xi’an	11,379	87 (0.8)	36 (0.3)	45.45	< 0.001
Shanghai	12,005	84 (0.7)	32 (0.3)	46.44	< 0.001
Nanjing	11,121	30 (0.3)	12 (0.1)	16.05	< 0.001
Wuhan	12,135	136 (1.1)	61 (0.5)	69.31	< 0.001
Guangzhou	10,019	462 (4.6)	220 (2.2)	234.19	< 0.001
Fuzhou	11,704	195 (1.7)	75 (0.6)	110.63	< 0.001
Kunming	11,217	331 (3.0)	197 (1.8)	119.52	< 0.001

Stunting was defined as < − 2 SD from median for length/height-for-age. *WHO* World Health Organization, *SD* standard deviation

### Underweight prevalence in 2016

The total prevalence of underweight based on the Chinese criteria was 2.0%, with boys 2.0% and girls 2.1% and urban 2.2% and rural 1.9% (Table 3). The underweight prevalence ranged from 1.5% to 2.7% among different age groups, from the lowest 0.7% in Nanjing to the highest 3.7% in Kunming. The underweight prevalence was lower based on the WHO criteria than based on the Chinese criteria in all the corresponding examined groups (Table 3).

**Table 3:** Prevalence of underweight, overweight and obesity in children under 7 years in nine cities of China based on the China and WHO criteria

Variables	Sample size	China criteria				WHO criteria				Chi-square	*P*
		Underweight	Normal weight	Overweight	Obesity	Underweight	Normal weight	Overweight	Obesity		
Total	110,491	2260 (2.0)	89,525 (81.0)	13,963 (12.6)	4743 (4.3)	1380 (1.2)	89,373 (80.9)	15,001 (13.6)	4737 (4.3)	1014.43	< 0.001
Gender											
Boys	57,918	1142 (2.0)	46,614 (80.5)	7331 (12.7)	2831 (4.9)	748 (1.3)	45,112 (77.9)	8731 (15.1)	3327 (5.7)	2321.20	< 0.001
Girls	52,573	1118 (2.1)	42,911 (81.6)	6632 (12.6)	1912 (3.6)	632 (1.2)	44,261 (84.2)	6270 (11.9)	1410 (2.7)	1164.76	< 0.001
Age (y)											
1–11 mon	17,327	474 (2.7)	14,799 (85.4)	1705 (9.8)	349 (2.0)	220 (1.3)	13,795 (79.6)	2762 (15.9)	550 (3.2)	1594.77	< 0.001
1–1.9	16,327	340 (2.1)	13,694 (83.9)	1950 (11.9)	343 (2.1)	118 (0.7)	13,026 (79.8)	2738 (16.8)	445 (2.7)	1214.00	< 0.001
2–2.9	13,669	271 (2.0)	11,428 (83.6)	1640 (12.0)	330 (2.4)	125 (0.9)	11,198 (81.9)	2007 (14.7)	339 (2.5)	482.89	< 0.001
3–3.9	15,453	348 (2.3)	12,485 (80.8)	2047 (13.2)	573 (3.7)	206 (1.3)	12,986 (84.0)	1763 (11.4)	498 (3.2)	546.61	< 0.001
4–4.9	19,931	373 (1.9)	15,819 (79.4)	2707 (13.6)	1032 (5.2)	229 (1.1)	16,597 (83.3)	2237 (11.2)	868 (4.4)	940.02	< 0.001
5–5.9	17,008	293 (1.7)	13,192 (77.6)	2343 (13.8)	1180 (6.9)	285 (1.7)	13,584 (79.9)	2037 (12.0)	1102 (6.5)	270.08	< 0.001
6–6.9	10,776	161 (1.5)	8108 (75.2)	1571 (14.6)	936 (8.7)	197 (1.8)	8187 (76.0)	1457 (13.5)	935 (8.7)	61.48	< 0.001
Area											
Urban	55,520	1195 (2.2)	45,336 (81.7)	6741 (12.1)	2248 (4.0)	725 (1.3)	45,310 (81.6)	7252 (13.1)	2233 (4.0)	539.05	< 0.001
Rural	54,971	1065 (1.9)	44,189 (80.4)	7222 (13.1)	2495 (4.5)	655 (1.2)	44,063 (80.2)	7749 (14.1)	2504 (4.6)	476.33	< 0.001
Region/cities											
Beijing	15,647	253 (1.6)	12,755 (81.5)	1850 (11.8)	789 (5.0)	108 (0.7)	12,775 (81.6)	1979 (12.6)	785 (5.0)	161.95	< 0.001
Harbin	15,270	353 (2.3)	12,106 (79.3)	1957 (12.8)	854 (5.6)	263 (1.7)	12,023 (78.7)	2138 (14.0)	846 (5.5)	116.57	< 0.001
Xi’an	11,377	269 (2.4)	9249 (81.3)	1453 (12.8)	406 (3.6)	204 (1.8)	9247 (81.3)	1512 (13.3)	414 (3.6)	61.66	< 0.001
Shanghai	12,005	127 (1.1)	9822 (81.8)	1547 (12.9)	509 (4.2)	76 (0.6)	9747 (81.2)	1700 (14.2)	482 (4.0)	80.30	< 0.001
Nanjing	11,121	79 (0.7)	8470 (76.2)	2031 (18.3)	541 (4.9)	39 (0.4)	8379 (75.3)	2161 (19.4)	542 (4.9)	66.12	< 0.001
Wuhan	12,134	209 (1.7)	9546 (78.7)	1872 (15.4)	507 (4.2)	132 (1.1)	9450 (77.9)	2009 (16.6)	543 (4.5)	124.63	< 0.001
Guangzhou	10,019	306 (3.1)	8473 (84.6)	961 (9.6)	279 (2.8)	166 (1.7)	8589 (85.7)	985 (9.8)	279 (2.8)	138.00	< 0.001
Fuzhou	11,702	253 (2.2)	9422 (80.5)	1420 (12.1)	607 (5.2)	147 (1.3)	9473 (81.0)	1478 (12.6)	604 (5.2)	98.82	< 0.001
Kunming	11,216	411 (3.7)	9682 (86.3)	872 (7.8)	251 (2.2)	245 (2.2)	9690 (86.4)	1039 (9.3)	242 (2.2)	217.55	< 0.001

Underweight was defined as < − 2 SD, overweight as ≥ + 1 SD but < + 2 SD, and obesity as ≥ + 2 SD from median for BMI-for-age. *BMI* body mass index, *WHO* World Health Organization, *SD* standard deviation

### Overweight and obesity prevalence in 2016

The total prevalence of overweight and obesity based on the Chinese criteria was 12.6% and 4.3%, respectively, with 12.7% and 4.9% for boys and 12.6% and 3.6% for girls (Table 3). The prevalence of overweight and obesity increased with age, with overweight increasing from 9.8% in the 1- to 11-month group to 14.6% in the 6- to 6.9-year group and obesity increasing from 2.0% to 8.7%. The overweight and obesity prevalence was 12.1% and 4.0% in urban areas and 13.1% and 4.5% in rural areas, respectively, varying from the lowest 7.8% in Kunming to the highest 18.3% and in Nanjing for overweight and from the lowest 2.2% in Kunming to the highest 5.6% in Harbin for obesity. The obesity prevalence based on the WHO criteria seemed to be close to that based on the Chinese criteria, such as overall 4.3% versus 4.3%, urban 4.0% versus 4.0% and rural 4.6% versus 4.5%; however, differences existed in gender, age and cities (Table 3).

## Discussion

**Fig. 5:**
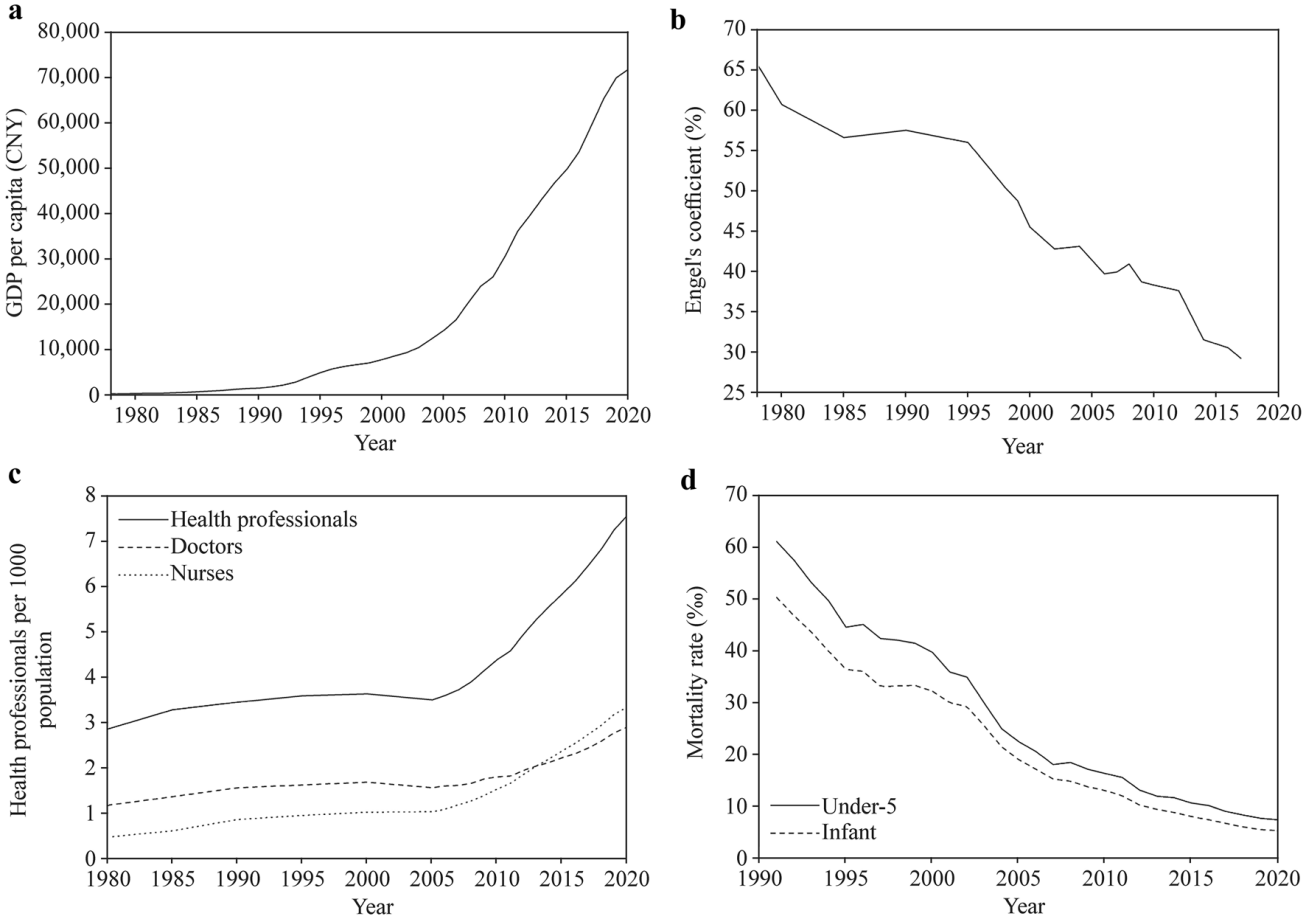
Secular trends in gross domestic product (GDP) per capita, Engel’s coefficient, health professionals per 1000 population, and infant and under-5 mortality rate in China, 1978–2020. **a** GDP; **b** Engel’s coefficient; **c** health professionals; **d** mortality rate

In the past few decades, China has achieved world-renowned socioeconomic progress, which has had a profound impact on various walks of life. GDP per capita increased from 385 Chinese Yuan (CNY) in 1978 to 72,000 CNY in 2020, Engel’s coefficient decreased from 65.9% in 1978 to 29.3% in 2017, health professionals rose from 2.85 per 1000 population in 1980 to 7.57 per 1000 population in 2020 with doctors from 1.17 to 2.90 and nurses from 0.47 to 3.34, infant mortality rate declined from 50.2‰ in 1991 to 5.4‰ in 2020 and under-5 mortality rate from 61.0‰ to 7.5‰, and these changing trends of economic and sociodemographic variables are shown in Fig. 5. During this period, children under 7 years old presented a positive rapid secular growth trend; furthermore, children from 7 to 18 years also exhibited a similar positive rapid secular trend [17, 18]. In general, this positive rapid secular change in Chinese children should mainly be attributed to rapid socioeconomic progress and improved health services.

Several notable features in the secular growth trend in China may have important public health implications. First, a slowing tendency was the latest and most prominent feature in the process of secular trend in Chinese children. Children’s health and nutrition strategies should be adjusted swiftly from focusing on physical growth level to promoting integrated early development. In addition, promoting socioeconomic development and improving people’s livelihood and health services was regarded as a powerful and replicable measure to ensure children’s growth potential. Second, the narrowing urban‒rural difference in height directly resulted from larger increments in rural children than urban children, especially in 2015. The urbanization construction of suburban rural areas has been accelerating and incorporating urban areas, especially in the last 10 years [3]. Perhaps urban‒rural disparities in children’s growth will further narrow or even disappear in the near future. Moreover, the narrowing urban‒rural difference further suggested that promoting socioeconomic development and urbanization construction was also regarded as an important measure to achieve equal development of urban, suburban and rural areas. Third, the growth level of children in cities of China has reached WHO child growth standards under 5 years since 2005. A systematic analysis showed that Chinese urban children under 5 years were the tallest in 141 low-income and middle-income countries in 2011, and Chinese rural children were put as the ninth tallest out of 141 countries with respect to rural height [19]. In general, children in cities of China have basically realized their growth potential and reached the standard of a well-nourished population, and children in rural regions have been rapidly catching up.

Under-nutrition in children is no longer the main nutritional concern in cities of China. In the nine survey cities, the prevalence of stunting and underweight was about 1% in 2016, regardless of the China or WHO criteria. Trend analysis from the series of the NSPGDC showed that child nutrition has entered a new development stage in China, with over-nutrition prevalence overtaking under-nutrition prevalence in Chinese cities since 2005 [20]. China has lifted all rural poor populations and regional overall poverty in 2022, and the problem of malnutrition in rural areas will be solved soon. However, considering that China is a large developing country with a vast territory and a large regional socioeconomic disparity, under-nutrition in rural children is still a major public health concern that cannot be ignored in some vulnerable regions and subgroups [21, 22], and child nutrition in rural areas, especially in poor rural areas, still needs to be given sustainable action.

Overweight and obesity in children is a serious public health problem in cities of China. Secular trends in BMI trajectories from the NSPGDC illustrated an alarming increase at the upper percentiles, which was the most direct evidence of an apparent increase in obesity prevalence in children. Data from the series of the NESSOC indicated a rapid increasing trend in obesity prevalence in children under 7 years of age in Chinese cities between 1986 and 2016 [11, 12]. Similarly, data from another national survey also illustrated the rapid increasing trend in obesity prevalence in children aged 7–18 years in China over the past decades [23]. Briefly, a rapid rise in obesity prevalence was the most noteworthy epidemic characteristic among children and adolescents in China in recent decades. In addition, focusing attention should be given to emerging epidemic features in that the overweight and obesity prevalence in rural areas increased faster and was even higher than that in urban areas. In 2010–2014, overweight and obesity showed a more rapid increase for children aged 7–18 years in rural areas than in urban areas [7, 23]. Recent epidemic features of overweight and obesity in children in China strongly support that obesity prevention and control in childhood should target not only urban children but also rural children.

Our study has several strengths. First, we used the earliest, large-scale national data to investigate long-term trends in height trajectories in Chinese children from 1975 to 2015. Second, we illustrated more comprehensive BMI trajectories by the 5th, 50th and 95th percentiles from 1985 to 2015. Third, we calculated and compared the updated prevalence of stunting, underweight, overweight and obesity using the latest NESSOC data based on both the China and WHO criteria. However, our study also had several limitations. First, the NSPGDC and its subproject (NESSOC) were undertaken in cities and did not cover rural areas, so the malnutrition prevalence in this present study may be inadequate in reflecting the situation in all of China. Second, our data only covered the 0–7-year part of the national studies rather than the whole 0–18-year range. Secular trends in height and weight status aged 7–18 years have been described elsewhere [6, 7, 24, 25]. Third, we illustrated the change in economic or sociodemographic variables in the general Chinese population; however, the change in the specific study population that may be more exact for exploring its association with children’s growth and nutrition was not publicly obtained for all the surveyed cities in the survey years.

In conclusion, in the process of a positive secular trend in China over the past few decades, the average height level of urban children under 7 years has reached WHO child growth standards since 2005 and presented a slowing tendency in the trend in 2005‒2015. The BMI trajectory highlighted an apparent increase in children at ages 3 years and older from 1985 to 2015, especially at the upper percentiles. More attention and efforts and public health interventions should be urgently made to combat overweight and obesity epidemics among preschool children. Our observed achievements and challenges from a rapidly developing China may be useful for both China and other rapidly developing countries or regions to revise and refine children’s health and nutrition strategies.

## Data Availability

The datasets used during the current study are not publicly available due to limitations of ethical approval involving the subject data and anonymity, but are available from the corresponding author on reasonable request.
